# Moral emotions in mental health: regulation and mediation

**DOI:** 10.3389/fpsyg.2025.1718674

**Published:** 2026-01-21

**Authors:** Angela Lu Wang

**Affiliations:** Steinhardt School of Culture, Education, and Human Development, New York University, New York, NY, United States

**Keywords:** cognitive neuroscience, mediation, mental health, moral emotions, regulation

## Abstract

Moral emotions—such as guilt, shame, compassion, and gratitude—play a central role in shaping psychological functioning and mental health. This mini review synthesizes recent empirical and theoretical research on the regulatory roles and mediating mechanisms of moral emotions in mental health outcomes. It is demonstrated that moral emotions activate both adaptive (e.g., cognitive reappraisal, self-compassion) and maladaptive (e.g., rumination, suppression) regulatory processes that significantly influence emotional resilience or vulnerability. Additionally, psychological flexibility, emotion regulation capacity, and cognitive mechanisms such as automatic negative thoughts are identified as key mediators linking moral emotions to conditions including anxiety, depression, and subjective wellbeing. Recent findings from cognitive neuroscience are also integrated, highlighting the ventromedial and dorsomedial prefrontal cortices, as well as other structures, as neural correlates involved in the regulation of moral emotions. This review advances understanding of how moral emotions modulate mental health and to inform emotion-focused interventions.

## 1 Introduction

Moral emotions—such as guilt, shame, compassion, and gratitude—represent a specialized category of affective experiences critically involved in regulating social behaviors and interpersonal relationships (Tangney, 1999). Guilt and shame are self-conscious emotions that are primarily self-evaluative and linked to internal moral standards, often prompting reparative or avoidant behaviors (Tangney et al., 2007), while compassion and gratitude are other-oriented/prosocial emotions that are outwardly focused, fostering social connection and reinforcement (Goering et al., 2024). These emotions inherently reflect social values and ethical standards, playing an essential role not only in guiding moral judgments and actions but also profoundly influencing individual mental health outcomes (Tangney et al., 2007; Haidt, 2003; Goering et al., 2024). Recent advances in psychology suggest that the ways individuals manage moral emotions significantly affect psychological adaptation, resilience, and overall wellbeing (Rehman et al., 2024).

The regulatory pathways of moral emotions primarily involve the process of emotion regulation, through which individuals modify their emotional experiences, expressions, and physiological responses. Effective regulation strategies, such as cognitive reappraisal and acceptance, have been demonstrated to promote psychological wellbeing, reduce emotional distress, and enhance resilience in the face of adversity (Troy et al., 2018; Han et al., 2023; Shum et al., 2024). Conversely, maladaptive strategies, including rumination, suppression, and avoidance, have been associated with heightened psychological distress, anxiety, and depression (Visted et al., 2018; Miethe et al., 2023; Alawadhi et al., 2023). Moral emotions often trigger these regulatory processes by intensifying self-reflective thoughts and social comparisons, prompting individuals to employ adaptive or maladaptive coping strategies.

In addition to direct regulatory pathways, moral emotions exert their effects on mental health through complex mediating mechanisms involving various cognitive and social variables. For instance, moral disengagement—an individual's cognitive justification for unethical behavior—has been shown to mediate the relationship between moral emotions, such as guilt or shame, and psychological outcomes (Aftab and Malik, 2021). Further research indicates that factors like emotional self-efficacy, self-compassion, and emotional awareness significantly mediate the impact of moral emotions on psychological functioning and resilience (Rehman et al., 2024; Liu et al., 2022). Understanding these mediating variables provides deeper insight into how moral emotions indirectly shape mental health trajectories.

Despite the growing recognition of moral emotions' significance, systematic synthesis of evidence elucidating how these emotions regulate psychological wellbeing and the underlying mediating mechanisms remains limited. This review aims to bridge this gap by critically integrating recent research findings on the regulatory pathways and mediating mechanisms of moral emotions, thereby providing a coherent conceptual framework that highlights the psychological and neuroscientific processes involved. This review focuses on guilt, shame, compassion, and gratitude because together these emotions form a coherent, mechanism-relevant moral–affective system that spans both risk pathways and repair pathways in mental health. Existing reviews often examine moral emotions in silos: shame and guilt are typically treated as negative self-conscious predictors of internalizing symptoms, compassion is frequently discussed as a stand-alone intervention construct, and gratitude is commonly framed within positive psychology. In contrast, this review conceptualizes these four emotions as successive and interacting components of a moral process that links moral appraisal to emotion regulation and downstream psychopathology vs. recovery.

## 2 Methodology

This review synthesizes empirical and theoretical literature from the past three decades (1995–2025), with a particular emphasis on high-impact findings from 2020 onward to capture the most recent developments. Literature was identified through systematic searches in PubMed, PsycINFO, and Web of Science using keywords including “moral emotions,” “guilt,” “shame,” “compassion,” “gratitude,” “emotion regulation,” “mediation,” “mental health” and related neuroscientific terms.

Inclusion criteria were: (1) peer-reviewed journal articles in English; (2) studies examining the relationship between at least one of the focal moral emotions (guilt, shame, compassion, gratitude) and mental health outcomes (e.g., depression, anxiety, wellbeing); (3) studies investigating regulatory pathways (e.g., reappraisal, acceptance, rumination, suppression) or mediating psychological constructs (e.g., psychological flexibility, automatic thoughts); (4) human studies including behavioral, clinical, and neuroimaging research.

## 3 Research findings

### 3.1 Regulatory pathways of moral emotions in mental health

Self-conscious moral emotions like guilt and shame serve as internal signals that prompt regulatory behaviors aimed at realigning one's actions with moral standards. Guilt often leads to reparative efforts (e.g. apologies, amends), which restore moral identity and interpersonal trust—this pathway is associated with emotional relief and improved wellbeing. Shame, in contrast, tends to induce global self-devaluation and social withdrawal. When individuals use maladaptive regulation strategies such as rumination or avoidance, shame often contributes to increased distress and poorer mental health outcomes (Erbildim and Nweke, 2025). Moral emotions activate behavioral regulatory responses beyond direct reparation. The moral cleansing mechanism describes how guilt and shame motivate individuals toward prosocial or symbolic actions (e.g., volunteering, charitable acts) after moral transgressions, aiming to restore a positive moral self-image (West and Zhong, 2015). Such compensatory behaviors not only mollify guilt but reinforce moral identity and social belonging, thereby buffering mental distress.

Moral emotions influence mental health by engaging explicit and implicit emotion regulation strategies. For instances, guilt or shame can be reinterpreted in less threatening ways—e.g., “I made a mistake, but I can learn”—which reduces emotional intensity and supports adaptation (Zhu et al., 2024; Rodrigues et al., 2024). Simply naming one's moral emotion (e.g., writing “I feel guilty”) engages the ventrolateral prefrontal cortex (vlPFC) and down-regulates amygdala activity, diminishing autonomic arousal and subjective distress (Torre and Lieberman, 2018).

Emerging theoretical models cast moral emotions within broader, multi-level emotion regulation frameworks. The virtue-based psychosocial adaptation model (V-PAM) proposes that moral emotions support adaptive regulation not only through cognition but via integration of moral values and identity (e.g., compassion as a virtue enhancing resilience; Eisenberg, 2000; Kim et al., 2024). Multilevel regulatory practices—intrapersonal (self-reappraisal), interpersonal (seeking social repair), and contextual (helping behaviors)—are associated with better mental health. Individuals who flexibly deploy strategies across levels fare better during existential stressors, such as pandemics or communal crises (Zhu et al., 2024). Positive moral emotions trigger distinct regulatory pathways that foster psychological flourishing. Compassion toward others encourages prosocial engagement and shared emotional regulation, which strengthens social bonds and reduces interpersonal stress. Gratitude enhances wellbeing via both emotion regulation and social reinforcement. Practicing gratitude activates neural reward circuits, promoting positive affect and resilience to stress (Lee and Contreras, 2023; Rieck et al., 2024). Table 1 shows commonly discussed adaptive and maladaptive regulation strategies/pathways for the four moral emotions (guilt, shame, compassion, and gratitude) and their typical implications for mental-health outcomes.

**Table 1:** The regulation strategies/pathways for the studied moral emotions and their typical implications for mental-health outcomes.

**Moral emotion**	**Adaptive regulation strategies/pathways**	**Maladaptive regulation strategies/pathways**	**Typical mental-health implications**	**References**
Guilt	Behavior-focused appraisal → repair: problem-solving, making amends, apology/reparation, recommitment to values	Contextually maladaptive/generalized guilt → rumination, self-punishment, avoidance	More normative guilt can be protective when it motivates repair; maladaptive guilt variants show stronger links with depressive and anxiety symptoms	Tangney et al., 2007; Tilghman-Osborne et al., 2010; Shapiro and Stewart, 2011; Kim et al., 2011
Shame	Reappraisal → specificity, self-compassion/self-reassurance, and values-consistent disclosure in safe relationships	Global self-condemnation → concealment, withdrawal, submissive/avoidant coping	Shame tends to show stronger associations with depressive symptoms than guilt; shame is also associated with anxiety symptoms	Kim et al., 2011; Cândea and Szentagotai-Tătar, 2018; Laving et al., 2023
Compassion (incl. self-compassion)	Affiliative/soothing activation → emotion regulation: compassionate attention, understanding, and supportive action tendencies; increases self-reassurance and distress tolerance	Barriers such as fear of compassion or collapsing into empathic distress/overarousal without self–other boundaries	Compassion-based constructs and interventions are linked to improvements in outcomes such as self-criticism/self-reassurance and reductions in distress	Gilbert, 2014; Neff and Germer, 2013; Han and Kim, 2023; Leaviss and Uttley, 2015; Millard et al., 2023
Gratitude	Positive reappraisal + savoring → broaden coping: attention to benefits, meaning-making, and positive affect; social bonding via expressing appreciation	Undermined by negative attentional bias or pressured/inauthentic “gratitude”	Trait gratitude is linked to better wellbeing and lower distress; gratitude interventions show small-to-moderate average improvements in wellbeing outcomes (effects vary by design/population)	Wood et al., 2008, 2010; Cregg and Cheavens, 2021; Choi et al., 2025

### 3.2 Mediating mechanisms of moral emotions in mental health

Moral emotions do not operate in isolation; rather, their effects on mental health are often mediated through a range of psychological constructs and processes. These mediating mechanisms are essential to understanding how moral emotions influence mental health outcomes, including depression, anxiety, and overall wellbeing.

Emotion regulation is one of the most robust mediators linking moral emotions to psychological outcomes. (Karacul 2025) examined the mediating role of self-compassion in the relationship between shame (internal and external) and subjective wellbeing in young adults. Both external shame and internal shame negatively predicted self-compassion (β = −0.56/−0.50, *p* < 0.001) and subjective wellbeing (β = −0.27/−0.25, *p* < 0.001). Self-compassion was significantly related to subjective wellbeing (β = 0.41/0.38, *p* < 0.001). Self-compassion had a partial mediating role in the relationship between both internal and external shame and subjective wellbeing. Individuals with higher self-compassion experienced less psychological disruption from shame, suggesting that fostering self-compassion may neutralize the toxic effects of shame on mental wellbeing.

Wang et al. (2025) conducted a systematic review confirming that emotion regulation acted as a conduit through which moral emotions, especially shame and guilt, affect mental health. Adaptive strategies such as acceptance and cognitive reframing mitigate the adverse effects of moral distress, whereas maladaptive strategies like rumination and avoidance exacerbate them. Rehman et al. (2024) conducted a study among college teachers, and the findings demonstrated a significant positive impact of psychological resilience on mental health (β = 0.39, *p* < 0.001). Self-compassion (β = 0.18, *p* < 0.01) and cognitive reappraisal (β = 0.16, *p* < 0.01) positively mediated the relationship between psychological resilience and mental health, while expressive suppression (β = −0.12, *p* < 0.05) negatively mediated the relationship. Wong et al. (2025) employed a three-wave longitudinal design, finding that self-compassion and psychological flexibility significantly mediated the association between self-criticism (often linked with moral emotions like guilt and shame) and psychological distress. Participants with higher levels of self-compassion were more likely to exhibit emotional resilience and lower depressive symptoms over time.

**Figure 1:**
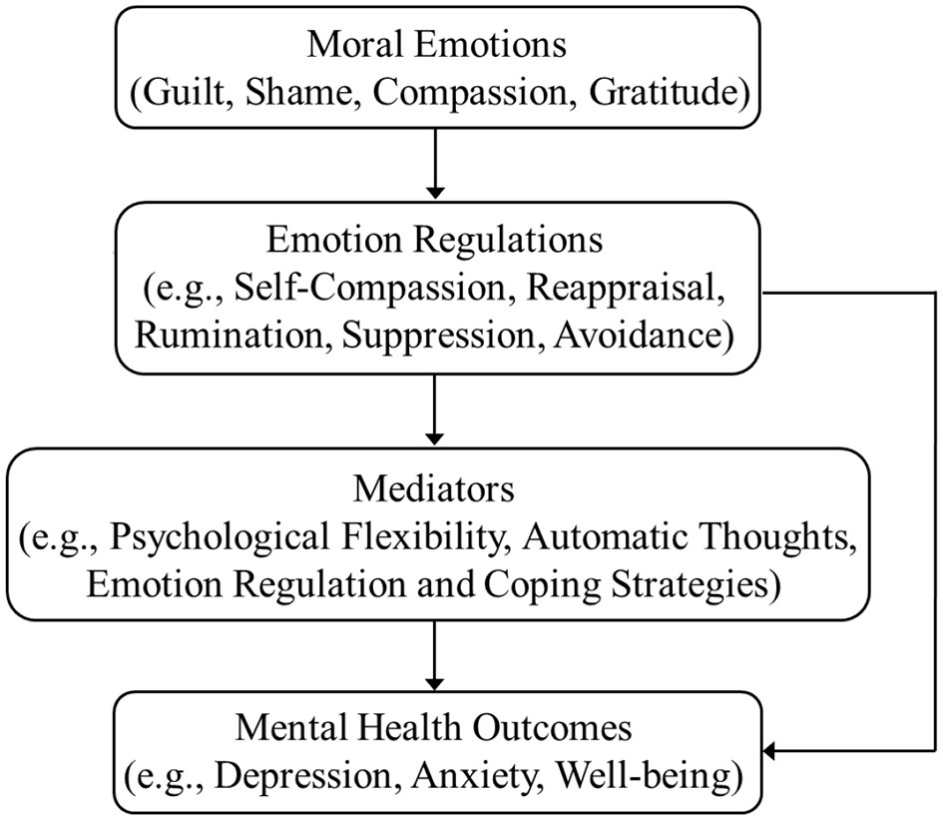
An inferred framework for the mediation between moral emotions and mental health outcomes.

In clinical populations, mediating mechanisms are especially relevant. Karlou and Karakasidou (2025) studied individuals with substance use disorders and identified self-compassion as a key mediator between emotion regulation difficulties and psychological distress. Individuals with higher self-compassion reported fewer symptoms of depression, anxiety, and stress, even in the presence of significant emotional dysregulation. This indicates that self-compassion not only mediates the effects of moral emotions but also provides a buffer against the negative consequences of emotional dysregulation in high-risk groups. Ren et al. (2025) demonstrated that automatic negative thoughts mediate the relationship between self-compassion and mental pain in individuals with major depressive disorder. The study emphasized that self-compassion was associated with reducing the frequency and intensity of automatic thoughts, thereby lessening the experience of mental pain. This chain of mediation illustrates how moral emotions, through self-compassion and automatic thoughts, can regulate internal cognitive processes to yield positive mental health outcomes. Based on the cited studies, an inferred framework for the mediation is illustrated in Figure 1.

### 3.3 Neuroscientific perspective

From a neuroscientific perspective, emerging evidence underscored the critical role of specific brain regions, particularly the ventromedial prefrontal cortex (vmPFC) and dorsomedial prefrontal cortex (dmPFC), in processing moral emotions and associated regulatory activities (Zahn et al., 2011; Hu and Jiang, 2014). Dysfunction or impairment in these regions has been linked with deficits in moral judgment, diminished social emotionality, impaired emotion regulation, and subsequently increased vulnerability to psychological disorders (Boes et al., 2011; Hiser and Koenigs, 2018). Therefore, exploring neurocognitive correlates of moral emotions enriches our understanding of their regulatory functions and potential psychopathological consequences.

The vmPFC and dmPFC are pivotal in processing and regulating moral emotions. vmPFC supports automatic, implicit regulation of moral emotions. Lesion studies demonstrated that damage to this region impairs guilt experience, trust, and moral decision-making, leading to more utilitarian responses in ethical dilemmas (Lockwood et al., 2024). dmPFC underlied explicit, self-referential emotional control, including reappraisal and moral reasoning processes (Xie et al., 2023; Jin et al., 2024). An fMRI meta-analysis shed light on neural dynamics associated with implicit emotion regulation, a process central to unintentional management of moral emotions. Patients with mood and anxiety disorders showed dysregulated activation in medial PFC regions (including BA9 and anterior cingulate cortex) during implicit emotional processing, suggesting a neural basis for maladaptive moral emotional responses. In contrast, explicit regulation strategies such as reappraisal typically engaged lateral PFC areas (vlPFC, dlPFC) in healthy individuals (Dalton et al., 2025). Neuroimaging showed that increased prefrontal control (vmPFC, vlPFC) over limbic responses is key for adaptive regulation—people with stronger prefrontal recruitment manage moral emotions more effectively and exhibit fewer anxiety or depressive symptoms (Motzkin et al., 2015; Rieck et al., 2024).

Intervention studies using neural modulation techniques further illustrate the causal role of prefrontal regions in emotion regulation. A focality-optimized transcranial direct current stimulation (tDCS) targeting the vmPFC improves implicit reappraisal, reducing negative emotion ratings and physiological arousal in highly anxious individuals, demonstrating the region's direct regulatory role (Gao et al., 2024). The interplay between affective valuation circuits and cognitive control mechanisms during positive social emotion upregulation is mediated via prefrontal networks, emphasizing top-down control in moral-affective contexts (Bezmaternykh et al., 2025).

There are limitations in the current researches which heavily focus on prefrontal cortex. This creates a narrow view and may undervalue the integrated, network-based nature of emotional processing, where other key structures (like the amygdala, anterior cingulate cortex, and insula) play essential roles in the generation and regulation of moral emotions. A few studied mentioned the connection between prefrontal cortex and other structures. Hiser and Koenigs (2018) reported that vmPFC was critical for the generation and regulation of negative emotion, through its interactions with amygdala, bed nucleus of stria terminalis, periaqueductal gray, hippocampus, and dorsal anterior cingulate cortex. Motzkin et al. (2015) reported that, relative to neurologically healthy comparison subjects, the vmPFC lesion patients exhibited potentiated amygdala responses to aversive images as well as elevated rest-state amygdala functional connectivity. Nevertheless, the connection between neural findings and clinical implications needs further elaboration.

## 4 Conclusion

This review synthesized findings across psychological theory, empirical research, and cognitive neuroscience to construct a comprehensive understanding of how moral emotions influence mental health through both regulatory and mediating pathways. The conclusions are: (1) moral emotions play a central regulatory role by activating a spectrum of emotional responses—ranging from adaptive strategies such as reappraisal and self-compassion to maladaptive responses like rumination and suppression; (2) mediators such as emotion regulation, psychological flexibility, and cognitive processes provide crucial explanations for how moral emotions affect mental health outcomes; (3) cognitive neuroscience findings underscore the role of specific brain structures—particularly the ventromedial and dorsomedial prefrontal cortices, as well as other structures—in processing and regulating moral emotions.

Future research should continue to explore the longitudinal and causal dynamics of these mechanisms, investigate individual and cultural differences in moral emotional processing, and develop targeted therapies that integrate emotion-focused and brain-based strategies. By advancing our understanding of moral emotions in mental health, we can move toward more precise, compassionate, and effective psychological care.
